# Progression of Pulmonary Emphysema and Continued Increase in Ectodomain Shedding of Cell Adhesion Molecule 1 After Cessation of Cigarette Smoke Exposure in Mice

**DOI:** 10.3389/fcell.2018.00052

**Published:** 2018-05-28

**Authors:** Aritoshi Ri, Man Hagiyama, Takao Inoue, Azusa Yoneshige, Ryuichiro Kimura, Yoshinori Murakami, Akihiko Ito

**Affiliations:** ^1^Department of Pathology, Kindai University Faculty of Medicine, Osaka-sayama, Japan; ^2^Division of Molecular Pathology, Institute of Medical Science, University of Tokyo, Tokyo, Japan

**Keywords:** pulmonary emphysema, animal model, histopathology, cell adhesion molecule 1, ectodomain shedding, ex-smoker

## Abstract

Pulmonary emphysema usually arises in cigarette smokers, and often progresses after smoking cessation and even in ex-smokers. Lung-epithelial cell adhesion molecule 1 (CADM1), an immunoglobulin superfamily member, is extracellularly shed to produce a proapoptotic C-terminal fragment (CTF) within the cell and contribute to the development of emphysema. Here, we made an ex-smoker model using C57BL/6 mice; mice (6-week-old; 5 mice per group) were exposed to passive smoke of eight cigarettes twice a day 5 days a week until 18 weeks of age, and were then left untreated until 30 weeks of age. We calculated the mean linear intercept (Lm) and the alveolar septal thickness in the lung histologic sections to estimate the alveolar space dilatation. At 18 weeks of age, Lm was marginally enlarged (*P* = 0.023) with a marked increase in the septal thickness (*P* < 0.001) in comparison with age-matched control mice (5 mice per group), while at 30 weeks, the increase in Lm was much more prominent (*P* = 0.006) and the septal thickness was normalized, suggesting that emphysema progressed with septal remodeling during smoking cessation. Western blot analyses of the lungs were performed for CADM1, a possible CADM1 sheddase ADAM10, an epithelial marker pan-cytokeratin, and a myofibroblastic marker α-smooth muscle actin to estimate the expression levels of CTF and ADAM10 per epithelial cell and the levels of pan-cytokeratin and αSMA per tissue. CADM1 shedding was increased in the treated mice than in control mice at both ages, in association with an increase in the CTF level at 30 weeks (*P* = 0.021). In total of the treated and control mice of 30 weeks of age, Lm was positively correlated with the CTF and ADAM10 levels, and pan-cytokeratin was negatively correlated with CTF, suggesting an involvement of CADM1 shedding in emphysema progression. Positive correlations were also found between CTF and ADAM10, and between ADAM10 and αSMA, suggesting that increased septal myofibroblasts might be involved in increased CADM1 shedding. Taken together, persisting increase in ectodomain shedding of CADM1 appeared to contribute to the progression of emphysema in ex-smokers, and might be accounted for by alveolar septal remodeling.

## Introduction

Pulmonary emphysema is a representative chronic obstructive pulmonary disease (COPD) characterized by alveolar wall destruction, resulting in enlarged airspaces and loss of surface area for gas exchange without fibrosis (Travis et al., 2002; Tomashefski, 2008). It is generally accepted that cigarette smoking is the most important risk factor for the development and progression of this disease. Cigarette smoking upsets the normal balance between proteases and antiproteases by producing particulates and oxidative stress on the lung that result in an influx of inflammatory cells (Tomashefski, 2008). Both neutrophils and macrophages are present in increased amounts in the lungs of smokers, and produce elastase and metalloproteinases, respectively (Tomashefski, 2008). These enzymes degrade elastin and other matrix components of the alveolar septum, resulting in alveolar destruction (Tomashefski, 2008). Therefore, smoking cessation is primarily important for avoiding or reducing the progression of emphysema (Rennard and Daughton, 2000). However, it is reported that once COPD is initiated, the pulmonary inflammation response continues and the enlarged alveolar airspace cannot be reversed following smoking cessation (Gamble et al., 2007; Braber et al., 2010).

We recently found lung-epithelial cell adhesion molecule 1 (CADM1), also known as tumor suppressor in lung cancer 1 (TSLC1) and nectin-like molecule 2 (Necl-2), to be involved in the development of emphysema (Mimae et al., 2014; Hagiyama et al., 2015). CADM1 is a member of the immunoglobulin superfamily, is expressed on bronchiolar and alveolar epithelial cells, and mediates intercellular adhesion between these cells (Ito et al., 2003; Shingai et al., 2003). This membrane-spanning glycoprotein is composed of three extracellular Ig-like domains, a single transmembrane region, and a short carboxy-terminal intracytoplasmic tail with a protein 4.1 interaction sequence and a PDZ type II domain-binding motif (Murakami, 2005). CADM1 is enzymatically cleaved at one of two sites in its ectodomain, yielding two membrane-associated C-terminal fragments, αCTF and βCTF (Nagara et al., 2012). One of the enzymes responsible for this ectodomain shedding is ADAM10 (Nagara et al., 2012). We found that CADM1 shedding increases in emphysematous lungs, and αCTF contributes to apoptosis of lung epithelial cells by localizing in mitochondria (Mimae et al., 2014). Considering that cigarette smoke is by far the most common etiologic agent for emphysema (Travis et al., 2002), oxidants in cigarette is likely to act as a critical inducer of CADM1 ectodomain shedding. However, our previous study revealed that ectodomain shedding of lung-epithelial CADM1 increased in smokers even after they had refrained from smoking for more than 1 month (Mimae et al., 2014). Therefore, oxidants seem not to promote CADM1 shedding through its direct, ongoing action. Among the clinical features of emphysema is a chronically progressive, irreversible disease (Tomashefski, 2008). This feature indicates defect of alveolar regeneration in the involved area. It is shown that repair functions of alveolar septal fibroblasts are impaired in patients with emphysema (Plantier et al., 2007; Togo et al., 2008). Nonetheless, alveolar septal remodeling appears to occur in the involved area, in association with the emergence of myofibroblasts that express alpha smooth muscle actin (αSMA) (Hallgren et al., 2012; Karvonen et al., 2013). We speculate that altered remodeling of the alveolar septum may be involved in increased CADM1 shedding in emphysematous lungs after smoking cessation.

In the present study, we attempted to make an ex-smoker model by exposing C57BL/6 mice to passive smoke for 12 weeks and then leaving them untreated for another 12 weeks. We histologically examined the lungs to confirm the development of emphysematous alveolar space dilatation. We also performed Western blot analyses of the lungs using antibodies against CADM1, ADAM10, an epithelial marker pan-cytokeratin, and a myofibroblastic marker αSMA. Correlations among the expression levels of these molecules and their correlations with airspace enlargement were analyzed. We finally examined whether H_2_O_2_, a representative oxidant, and neutrophil elastase could cause ectodomain shedding of lung epithelial CADM1.

## Materials and methods

### Ethical approval

This study was carried out in accordance with the recommendations of the *Guide for the Care and Use of Laboratory Animals* of the US National Institutes of Health (8th edition, revised in 2011). The protocol was approved by the Institutional Animal Experimentation Committee of Kindai University Faculty of Medicine (approval numbers KAME-26-062 and 23-022).

### Animals and experimental groups

**Figure 1:**
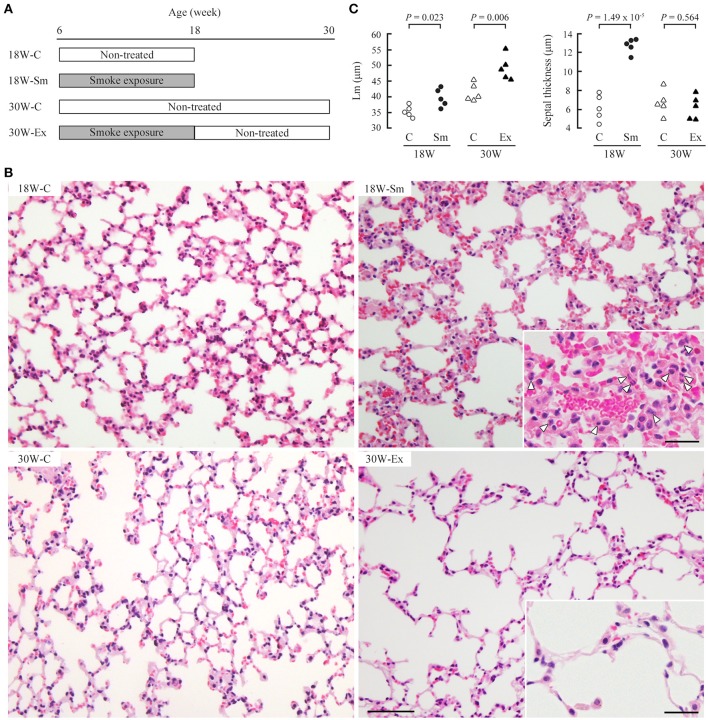
Development of emphysematous lesions in ex-smoker mice. **(A)** Male 6-week-old C57BL/6 mice were allocated randomly into four groups (five mice per group). 18 and 30W-C mice were left untreated and were sacrificed at the age of 18 and 30 weeks, respectively. 18W-Sm and 30W-Ex mice were exposed to cigarette smoke for 12 weeks, and 18W-Sm mice were sacrificed after the final exposure. 30W-Ex mice were thereafter left untreated until the age of 30 weeks. **(B)** Paraformaldehyde-fixed, paraffin-embedded lung sections were stained with hematoxylin and eosin. Representative histological images of the lung sections are shown for each mouse group. Bar = 200 μm. Magnified views are inserted to improve visibility of inflammatory cells (depicted by arrowheads). Bar = 25 μm. **(C)** Mean linear intercept (Lm; left) and alveolar septal thickness (right) of each mouse were plotted as dots, and statistical significance was analyzed between the two groups of the same age. *P*-values by paired Student's *t*-test are shown.

Male 4-week-old C57BL/6 mice were purchased from Japan SLC, Inc. (Hamamatsu, Japan), and were kept in specific pathogen-free conditions at a constant temperature and humidity, with *ad libitum* feeding. A total of 20 male 6-week-old mice were randomized into two groups (10 mice per group) (Figure 1A). Mice of the first group were left untreated, and five mice were randomly selected and sacrificed at 18 weeks of age (18W-C) and the remaining five were sacrificed at 30 weeks of age (30W-C). Mice of the second group were exposed to cigarette smoke for 12 weeks (until 18 weeks of age) according to the procedures described in the next paragraph. Five mice were randomly selected and sacrificed 1 h after the final exposure (18W-Sm). The remaining five mice were left untreated (exposed to room air) until 30 weeks of age, and were then sacrificed (30W-Ex).

*Cadm1*-deficient 129Sv/C57BL/6 strain mice (*Cadm1*-/-), which lack the entire exon 1 of the Cadm1 gene located on #9 autonomic chromosome, were generated as described previously (Yamada et al., 2006), and were backcrossed to wild-type C57BL/6 mice more than ten times. Littermates with three different *Cadm1* genotypes were obtained by mating the male and female *Cadm1*+/– mice. The lungs were removed at 11 weeks of age.

### Exposure to cigarette smoke

A closed box (W45 × D35 × H35 cm) was made of acrylic plates, and was equipped with a mesh flat acrylic floor at the middle portion of the vertical axis. The box was placed in a draft chamber, and 10 C57BL/6 mice (6-week-old, male) were placed on the mesh floor and were exposed to passive smoke by burning eight cigarettes (Marlboro; tar 12 mg, nicotine 1 mg per cigarette) at the bottom of the box until they burnt out (it took approximately 1 h). During this smoke exposure, the oxygen concentration was continuously monitored with an oximeter (OXY-1; Ichinen Jikco, Tokyo, Japan); when the concentration decreased below 20%, the chamber was opened for a while so as to normalize the concentration. This exposure treatment was repeated twice a day (in the morning and evening), 5 days a week (from Monday to Friday) for 12 weeks (until 18 weeks of age); five mice (18W-Sm) were sacrificed, and five of the rest (30W-Ex) were then left untreated for another 12 weeks (until 30 weeks of age). Numbers and concentrations of suspended particulates in the box during smoke exposure were measured using an air particle counter (MET ONE HHPC 6+; Beckman Coulter, Indianapolis, IN, USA) and a digital dust indicator (LD-5R; Sibata Scientific Technology, Tokyo, Japan). Representative monitoring measurements are shown in Table S1. Concentrations of total particulates in the present study were nearly constant during the treatment and approximately 17 mg/m^3^; this value indicates that the present treatment condition was mild when compared with those in similar past studies (Leberl et al., 2013).

### Histological and morphometric analyses

The lungs were excised immediately after sacrifice and fixed by immersion and intratracheal infusion with 4% paraformaldehyde, then embedded in paraffin. The middle portion of each lung lobe was cut along the longitudinal axis into 5-μm thick sections, and after deparaffinization, were stained with hematoxylin and eosin. Alveolar airspace enlargement was assessed according to the well-established method (Thurlbeck, 1967) with some modifications. The stained sections were observed through a light microscope at a magnification of ×200, and randomly selected 5 fields of view were captured. Six and eight lines were drawn in the horizontal and vertical directions, respectively, at an interval of 100 μm in each photomicrograph. On each line, the length of the portion crossing the alveolar airspace between two neighboring alveolar walls was measured. The lengths of all the portions on all lines of the five pictures were summed and divided by the total number of alveolar airspace, then expressed as the mean linear intercept (Lm) for the mouse. Lm and standard error were calculated from the Lms of five mice for each experimental group. In each of the lung section photomicrographs (×200), 50 sites were randomly selected on the alveolar septum and were measured for their thickness, and the mean was calculated from all measurements of the five photomicrographs for each mouse. The mean and standard error were calculated from the means of five mice, and was expressed as alveolar septal thickness for each experimental group.

Some lung sections were stained immunohistochemically as we described previously (Ito et al., 2003; Takashima et al., 2017). Briefly, deparaffinized sections were autoclaved for 20 min at 121°C in 10 mM citrate buffer (pH 6.0). Endogenous peroxidase activity was blocked by immersion in methanol containing 0.3% hydrogen peroxide for 5 min. Nonspecific Ig binding in the sections was blocked with 2% bovine serum albumin (BSA) in phosphate-buffered saline (PBS). Then the sections were reacted with the rabbit polyclonal anti-CADM1 antibody in PBS containing 2% BSA. Second antibody and signal enhancement reactions were performed using a Histofine Simple Stain kit (Nichirei Biosciences, Tokyo, Japan). Color was then developed with aminoethylcarbazole (ImmPACT™ AEC; Vector Laboratories, Burlingame, CA, USA), and the nuclei were counterstained with hematoxylin.

### Cell culture

RLE-6TN cells (lot no. 59111690), a rat lung epithelial cell line with characteristics of alveolar type II cells, were purchased from the American Type Culture Collection (Rockville, MD, USA). All experiments using this cell line were performed within 4 months after resuscitation. RLE-6TN cells were grown in Ham's F12 medium containing 2 mM L-glutamine (Gibco, Carlsbad, CA, USA) supplemented with 10% fetal bovine serum, 10 μg/ml bovine pituitary extract (PromoCell, Heidelberg, Germany), 5 μg/ml insulin (Gibco), 2.5 ng/ml insulin-like growth factor (Sigma-Aldrich, St. Louis, MO, USA), 1.25 μg/ml transferrin (Gibco), and 2.5 ng/ml epidermal growth factor (Sigma-Aldrich, St. Louis, MO, USA) as we described previously (Hagiyama et al., 2015). H_2_O_2_ (Wako Pure Chemical Industries, Osaka, Japan) was added to the semiconfluent culture of RLE-6TN cells at a concentration of 440 μM, according to the procedures published previously (Kim et al., 2014). After 4 or 8 h, H_2_O_2_ was removed by medium replacement, and the cultures were continued for 2 or 3 days. In another experiment, elastase (porcine pancreas; Worthington, Lakewood, NJ, USA) was added at a concentration of 1 unit/ml to the semiconfluent culture of RLE-6TN cells grown in the Ham's F12 medium same as described above except lacking fetal bovine serum. After 1 h, cells were subjected to Western blot analyses.

### CADM1 shedding induction in the excised lungs

Experiments were performed essentially according to published procedures (Han and Ziegler, 2013). Briefly, immediately after the lung was removed from male 6-week-old C57BL/6 mice, 2 ml of PBS alone or PBS containing either elastase (1 unit/ml) or a mixture of PMA (200 nM; Wako Pure Chemical Industries) and trypsin (0.0125% w/v; Wako Pure Chemical Industries) was intratracheally injected into the lung using a syringe attached to a 27-gauge needle. After ligation of the tracheal proximal cut end, the lung was placed in a CO_2_ incubator at 37°C for 30 min. The bronchoalveolar lavage fluid was aspirated using a syringe attached to a 27-gauge needle; approximately 250-μl fluid was collected per lung. The lung and the recovered fluid were subjected to Western blot analyses.

### Western blot analysis

Cells and mouse lungs were lysed in a buffer containing 50 mM Tris-HCl (pH 8.0), 150 mM NaCl, 1% Triton X-100 and 1 mM phenylmethylsulfonyl fluoride and were subjected to Western blot analyses as described in our previous report (Koma et al., 2008). The recovered bronchoalveolar lavage fluid was directly separated on SDS-PAGE gels. Two kinds of anti-CADM1 antibodies were used, which we previously generated; rabbit polyclonal antibody against the C-terminal peptide (eggqnnseekkeyfi) to detect full-length CADM1 and αCTF (Mimae et al., 2014), and chicken monoclonal antibody against the ectodomain (3E1) to detect the N-terminal fragment (NTF) released from the cell by CADM1 shedding (Furuno et al., 2005; Nagara et al., 2012). Other primary antibodies used in this study targeted ADAM10 (rabbit polyclonal; Millipore, Billerica, MA, USA), αSMA (mouse monoclonal clone 1A4; Dako, Santa Clara, CA, USA), E-cadherin (clone 36; BD Bioscience, San Jose, CA, USA), pan-cytokeratin (mouse monoclonal AE1/AE3; Dako), and β-actin (Medical & Biological Laboratories). Peroxidase-conjugated secondary antibodies were purchased from Amersham (Buckinghamshire, England). Immunoreactive band intensities were quantified using ImageJ software (National Institutes of Health, Bethesda, MD, USA), as described previously (Mimae et al., 2012).

### Statistical analysis

Differences between two experimental groups were analyzed by paired Student's *t*-test for Western blot intensities, CADM1 shedding rates, and morphological valuables. Correlations were analyzed using Spearman's rank test. All data sets of the X- or Y-axis of the scatter plots were analyzed using the Shapiro-Wilk normality test, and their data distributions were confirmed to be non-parametric (*P* ≤ 0.05). A *P* ≤ 0.05 was considered to indicate statistical significance.

## Results

### Development of emphysematous lesions in ex-smoker model mice

Ten C57BL/6 mice (6-week-old) were placed in a closed acrylic box chamber and were exposed to passive smoke of eight cigarettes (it took approximately 1 h) twice a day. This treatment was repeated 5 days a week for 12 weeks (until 18 weeks of age); five mice (18W-Sm) were sacrificed, and five of the rest (30W-Ex) were then left untreated for another 12 weeks. Figure 1B shows representative lung histology of the two group mice and age-matched control mice (18W-C and 30W-C). We calculated the mean linear intercept (Lm) in these sections to estimate the alveolar space dilatation. Lm was significantly larger in both 18W-Sm and 30W-Ex mice when compared with the corresponding age-matched mice, though the significance in 18W-Sm was marginal (*P* = 0.023 *vs*. *P* = 0.006 in 30W-Ex) (Figure 1C). Histological analyses also revealed that the alveolar septum was markedly thickened in 18W-Sm mice, in association with considerable infiltration of inflammatory cells including neutrophils (Figure 1B, insets). In contrast, the septum of 30W-Ex mice was as thin as that of control mice, and contained small numbers of inflammatory cells (Figure 1B, insets).

### Expression and correlation analyses of CADM1, ADAM10, αSMA, and Lm

**Figure 2:**
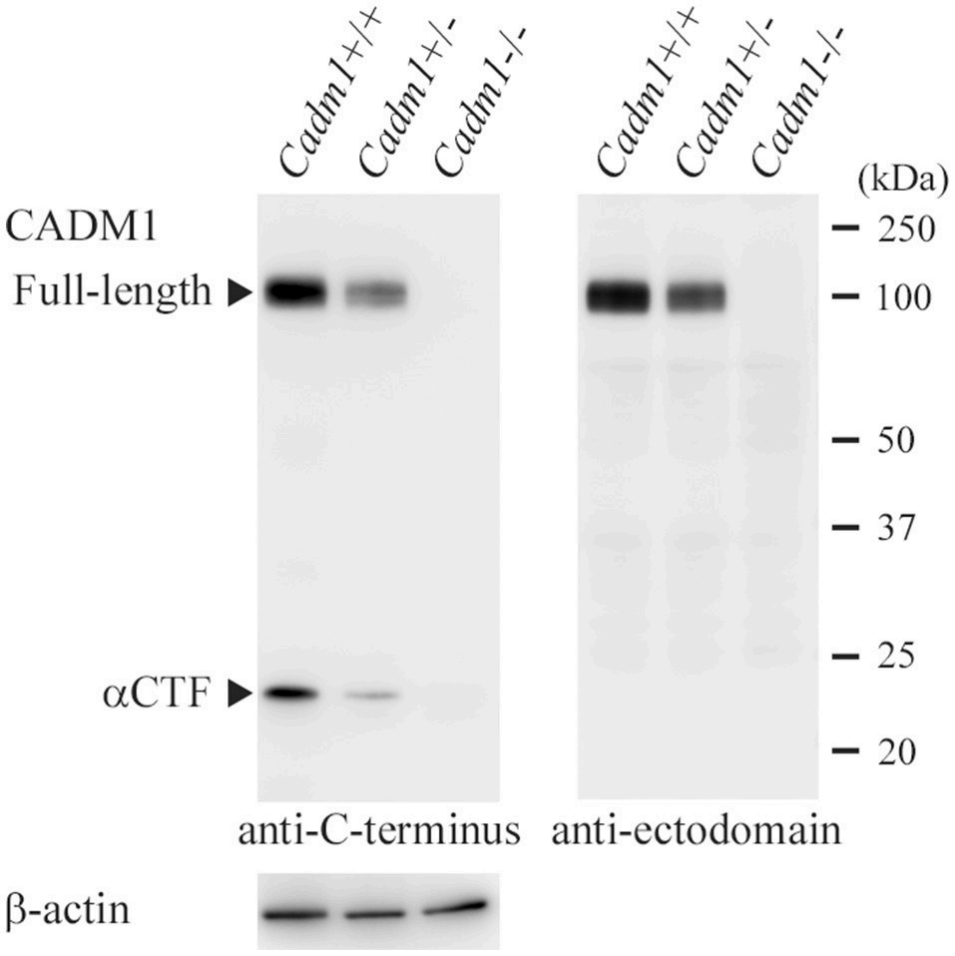
Western blot analyses of the lungs from littermate mice with different *Cadm1* genotypes. Protein was extracted from the lungs of littermate mice with different genotypes, *Cadm1*+/+, +/-, and -/-, and was subjected to Western blot analyses using antibodies against the C-terminus (left) and ectodomain (right) of CADM1. Arrowheads indicate bands corresponding to the full-length and αCTF forms of CADM1. The blot was reprobed with an anti-β-actin antibody to indicate the amount of protein loading per lane.

**Figure 3:**
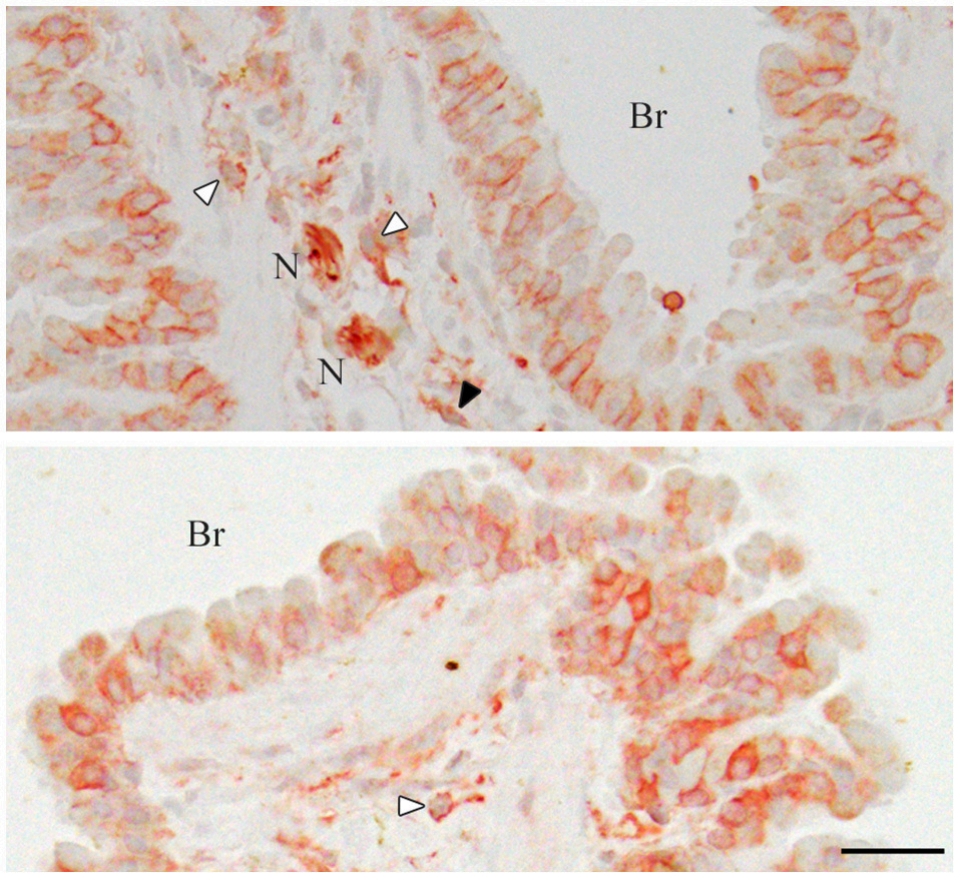
Immunohistochemistry of the mouse lungs for CADM1. The lung sections were stained immunohistochemically with an anti-CADM1 antibody and counterstained with hematoxylin. Representative results are shown; upper, 30W-C and lower, 30W-Ex. White and black arrowheads indicate mast cells and a possible fibroblast, respectively. Br, bronchus; N, nerve. Bar = 25 μm.

In order to check whether our rabbit polyclonal anti-CADM1 C-terminus antibody specifically recognizes CADM1 in the lung, we prepared from the lung lysates from littermate mice with three different genotypes *Cadm1*+/+, +/-, and -/-, and subjected them to Western blot analyses. The antibody did not yield any specific immunoreactive bands in the *Cadm1*-/- lung, but detected the full-length CADM1 and αCTF in the *Cadm1*+/- lung at just about half levels of those in the *Cadm1*+/+ lung (Figure 2). Anti-CADM1 ectodomain antibody 3E1 detected only the full-length CADM1 in *Cadm1*+/+ and +/- mouse lungs as strong as did the C-terminal antibody (Figure 2). These results indicate that the C-terminal antibody is specific to CADM1 at least in the mouse lung lysates. In addition, we performed immunohistochemical analyses of the lung sections using the anti-CADM1 C-terminus antibody. CADM1 positivity was detected mainly in epithelial cells and additionally in the nerves, mast cells and probably fibroblasts present in the connective tissue around the bronchus; the CADM1-positive non-epithelial cells were much smaller in number than CADM1-positive epithelial cells (Figure 3). Therefore, we considered that CADM1 expressions detected in Western blots of the lung lysates should be mainly derived from lung epithelial cells.

**Figure 4:**
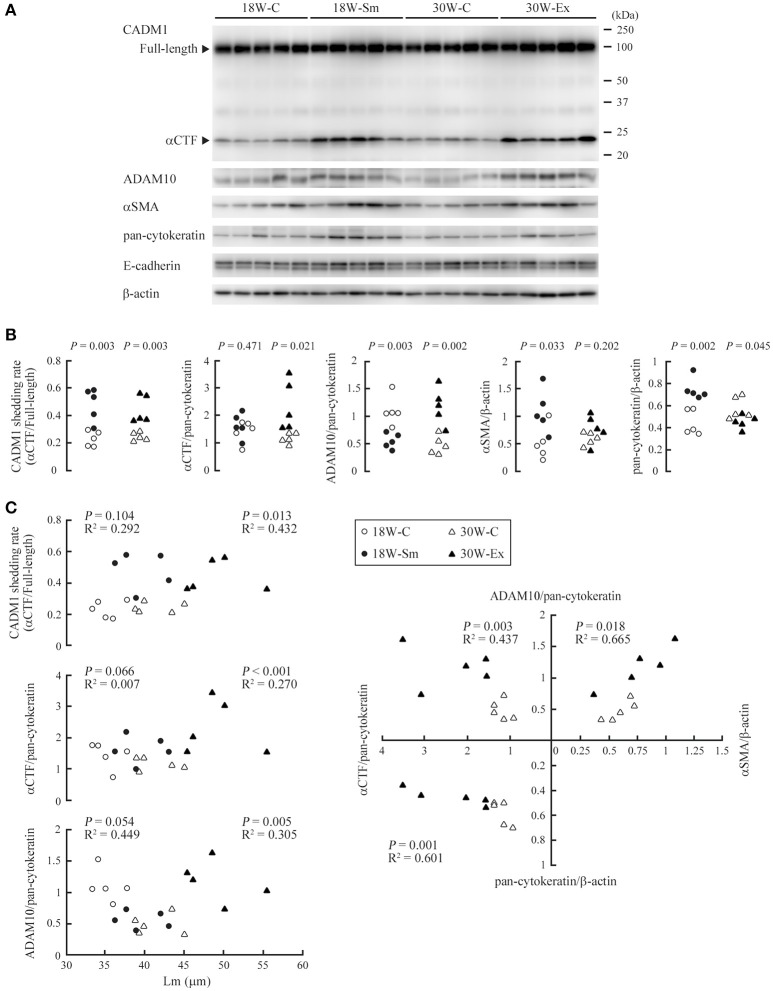
Expressions of CADM1 and related molecules in ex-smoker model mouse lungs and their mutual correlations. **(A)** Protein was extracted from the lungs of 20 mice, each allocated to one of the four groups (18W-C, 18W-Sm, 30W-C, and 30W-Ex), and was subjected to Western blot analyses using an anti-CADM1 antibody. Arrowheads indicate bands corresponding to the full-length and αCTF forms of CADM1. The blot was reprobed with an anti-ADAM10, anti-αSMA, anti-pan-cytokeratin, and anti-E-cadherin antibody. The blot was also probed with an anti-β-actin antibody to indicate the amount of protein loading per lane. **(B)** CADM1 ectodomain shedding rates (amount of αCTF relative to full-length CADM1), αCTF and ADAM10 levels per lung epithelial cell (relative to pan-cytokeratin), and αSMA and pan-cytokeratin levels per tissue (relative to β-actin) are plotted as dots in each group. Statistical significance between the two groups of the same age was analyzed using paired Student's *t*-test. *P*-values are shown. **(C)** The CADM1 shedding rate, αCTF and ADAM10 level per epithelial cell are shown in the upper three scatter plots with Lm. In the right graph, the αCTF and ADAM10 levels per epithelial cell, and αSMA and pan-cytokeratin levels per tissue are scatter plotted with each other. Correlations and statistical significance were analyzed using Spearman's rank test. *P*-values and *R*^2^ are shown.

We prepared tissue lysates from the lungs of 18W-Sm, 30W-Ex and age-matched control mice, and subjected them to Western blot analyses using antibodies against CADM1, ADAM10, αSMA, pan-cytokeratin, and E-cadherin (Figure 4A). We estimated the expression levels of αCTF and ADAM10 per epithelial cell by normalizing to pan-cytokeratin, because cytokeratins are widely used as an epithelial marker and less changes their expression levels in the lung of smokers or COPD patients (Milara et al., 2013). The CADM1 shedding rate (quantitative ratio of αCTF to full-length) was significantly higher in both 18W-Sm and 30W-Ex mice, and the αCTF level was increased in 30W-Ex mice, when compared with age-matched control mice (Figure 4B). The ADAM10 level was decreased in 18W-Sm mice and increased in 30W-Ex mice (Figure 4B). We also estimated the expression levels of αSMA and pan-cytokeratin per tissue by normalizing to β-actin. The αSMA level was increased in 18W-Sm mice, but unchanged in 30W-Ex mice (Figure 4B). The pan-cytokeratin level normalized to β-actin was increased in 18W-Sm mice but decreased in 30W-Ex (Figure 4B), reconfirming that the lung histology of 30W-Ex mice, not 18W-Sm mice, was similar to human emphysema, where lung epithelial cells are decreased in number. In contrast, the E-cadherin level relative to pan-cytokeratin was decreased in 18W-Sm mice, not in 30W-Ex mice (Figure S1A), suggesting that the 12-week smoke exposure might have induced epithelial to mesenchymal transition in the lung, consistent with past studies (Sohal et al., 2011; Milara et al., 2013). We also performed similar analyses using E-cadherin as an epithelial marker, and showed the results in Figure S2 as a reference.

Various pairs of the calculated values were scatter plotted separately in dot graphs, and were analyzed for correlation. In these analyses, we took into consideration the relatively high ages of the mice (the older group is 30 weeks; even the younger, 18 weeks) as well as the relatively small number of mice examined per group (*n* = 5). When 18W-C and 30W-C mice were compared, Lm was significantly larger in 30W-C mice (35.4 ± 1.74 *vs*. 41.4 ± 2.73, *P* = 0.003; Figure 1C). We considered that this increase in Lm in the older mouse group might be explained by the same mechanism as that for 30W-Ex mice, i.e., age-dependent increase in CADM1 shedding. Likewise, 18W-C mice may have a larger Lm than younger mice. Therefore, we performed correlation analyses in the combination group of control and treated mice of the same age. The CADM1 shedding rate, and the αCTF and ADAM10 levels per epithelial cell were positively correlated with Lm at 30 weeks of age, but were not at 18 weeks of age (Figure 4C). At 30 weeks of age, the αCTF level per epithelial cell was negatively correlated with the pan-cytokeratin per tissue, and was positively correlated with the ADAM10 level per epithelial cell, which in turn was correlated with the αSMA level per tissue (Figure 4C). There was a strong correlation between the CADM1 shedding rate and the αCTF level per epithelial cell at 30 weeks of age (Figure S1B). These results are consistent with our previous hypothesis that CADM1 shedding produces αCTF, and increased αCTF contributes to the decrease in lung epithelial cells (Mimae et al., 2014).

### Hydrogen peroxide (H_2_O_2_), not elastase, induces ectodomain shedding of lung epithelial CADM1

**Figure 5:**
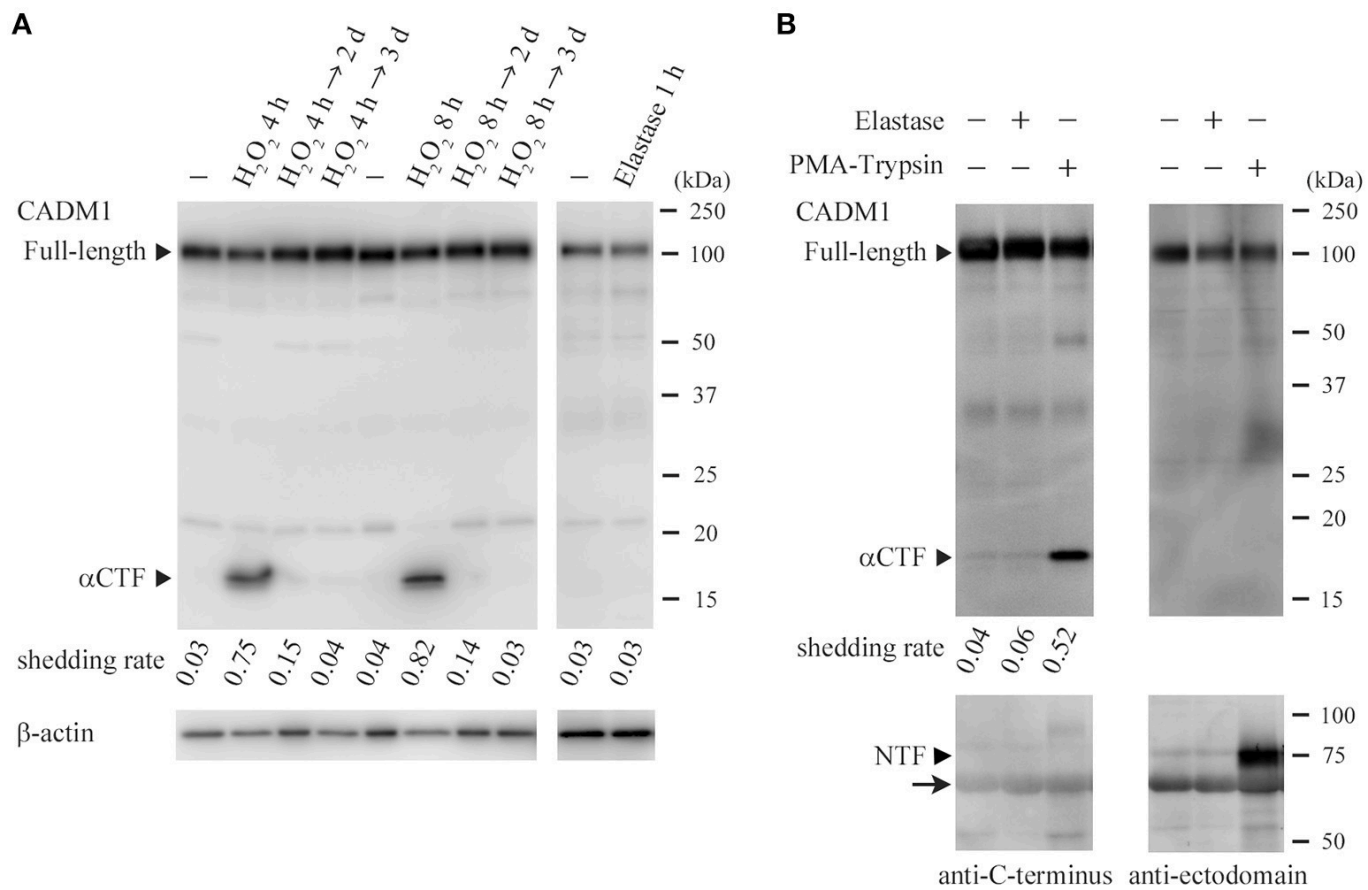
Induction of ectodomain shedding of lung epithelial CADM1 by H_2_O_2_, not by elastase. **(A)** RLE-6TN cells were treated with H_2_O_2_ (440 μM) for 4 or 8 h. After removal of H_2_O_2_ by medium replacement, cell cultures were continued for 2 or 3 days. Cell lysates were prepared before (-) and immediately after (H_2_O_2_ 4 or 8 h) the H_2_O_2_ treatment, and 2 (→ 2 days) or 3 (→ 3 days) days after the medium replacement, and were subjected to Western blot analyses using an anti-CADM1 antibody (left). RLE-6TN cells were also treated with elastase (1 unit/ml) for 1 h. Cell lysates were prepared before (-) and immediately after the treatment, and were analyzed by Western blotting (right). Arrowheads indicate bands corresponding to the full-length and αCTF forms of CADM1. The blot was reprobed with an anti-β-actin antibody to indicate the amount of protein loading per lane. CADM1 shedding rates are shown for each lane. **(B)** PBS alone (-) or PBS containing either elastase (1 unit/ml) or a mixture of PMA (200 nM) and trypsin (0.0125% w/v) was intratracheally injected into the freshly excised mouse lung, and after the bronchial ligation, the lung was incubated in a CO_2_ incubator for 30 min, then the bronchoalveolar fluid was collected. Protein was extracted from the fluid (lower) and the lung (upper), and was subjected to Western blot analyses using antibodies against the C-terminus (left) and the ectodomain (right; 3E1) of CADM1. Arrowheads indicate bands corresponding to the full-length, αCTF and NTF forms of CADM1. An arrow indicates background bands for albumin contained in the bronchoalveolar fluid.

As CADM1 shedding was seen in the lung of non-treated 18W-C mice, we considered that oxidative stress and inflammatory cell-derived proteases might upregulate CADM1 shedding constitutively or transiently in lung epithelial cells. We examined whether lung epithelial CADM1 was shed by H_2_O_2_, a representative reagent of oxidants, and elastase, a serine protease contained in neutrophil granules, using rat lung epithelial RLE-6TN cells, in which CADM1 was not shed under the standard culture condition. When the cells were treated with H_2_O_2_ for 4 or 8 h, CADM1 was shed at a shedding rate of 0.7–0.8 (Figure 5A). When the cultures were continued for 2 or 3 days after the removal of H_2_O_2_ by medium replacement, the shedding rate markedly reduced to trace levels (Figure 5A). Similar treatment of RLE-6TN cells with elastase did not induce CADM1 shedding at all (Figure 5A). Possible involvement of elastase in CADM1 shedding was also examined in near *in vivo* settings. Elastase was administered intratracheally to the mouse lungs immediately after being removed. Similar experiments were done using a mixed solution of PMA and trypsin, known as the CADM1 shedding inducer (Mimae et al., 2014). After 30 min of incubation, proteins from the lung and the bronchoalveolar lavage fluid were analyzed by Western blotting. The lung treated with the PMA-trypsin mixture contained a considerable amount of αCTF, and NTF released from the cell by shedding were clearly detected in the bronchoalveolar lavage fluid. In contrast, neither αCTF nor NTF was detected in the elastase-treated lungs (Figure 5B).

## Discussion

It is known that cigarette smoke-mediated inflammatory and oxidative responses are strain-dependent in mice (Guerassimov et al., 2004; Yao et al., 2008). Because the C57BL/6 strain is highly sensitive to chronic exposure of cigarette smoke (Yao et al., 2008), we considered this strain to be suitable for observing what histological changes, i.e., disease progression or tissue repair/regeneration, occurred in the lungs after cessation of smoke exposure. In the present study, C57BL/6 mice were exposed to passive smoke for 12 weeks (18W-Sm), and were then left untreated for 12 weeks (30W-Ex). At the end of 12 weeks of passive smoke exposure, the Lm increased with statistical significance, though marginal, and the alveolar septum was irregularly thickened with considerable inflammatory infiltration, probably reflecting the highly sensitive nature of this mouse strain. Mice after 12 weeks of smoking cessation had the lungs that were histologically very similar to human emphysema, featured by remarkable airspace enlargement with thin, free-floating alveolar septa, and decreased epithelial cells (Tomashefski, 2008). Considering the life span of laboratory mice, 12 weeks of smoke exposure and non-treatment represent approximately 12 years of smoking and smoking cessation in humans. The present mouse model appeared to be useful for examining the pathogenesis of emphysema arising in ex-smokers who are sensitive to cigarette smoke and quitted smoking years ago.

We found that CADM1 shedding was increased not only at the end of 12-week-long smoke exposure (18W-Sm), but also after the next 12 weeks of non-treatment (30W-Ex) in association with increased amounts of αCTF per epithelial cell. Increased CADM1 shedding was suggested to contribute to the development of emphysematous lesions in mice at both timepoints. We also found that the ADAM10 expression levels in the lungs were increased in 30W-Ex mice, but decreased in 18W-Sm mice. That is, the increased shedding at 30 weeks appeared to be attributable to ADAM10, but did not so at 18 weeks. At the end of 12-week-long smoke exposure, the alveolar septa were thickened in association with infiltration of numerus inflammatory cells including neutrophils. Neutrophil elastase is recognized to contribute to the development and progression of emphysema in humans and mice (Gardi et al., 1994; Cavarra et al., 2001; Tomashefski, 2008), but did not appear to be involved in CADM1 ectodomain shedding, according to the results of Figure 5. Alveolar macrophages are also known to be involved in smoking-induced emphysema (Tomashefski, 2008), but there was no substantial increase in their numbers at 12 weeks of smoke exposure (Figure 1B). Instead, we showed that H_2_O_2_ could induce CADM1 shedding transiently in lung epithelial cells. Oxidants contained in cigarette smoke may be responsible for the increased CADM1 shedding in 18W-Sm mouse lungs.

There was an apparent difference in the alveolar septal thickness between 18W-C and 18W-Sm, whereas the Lm was comparable. On the other hand, the Lm was much larger in 30W-Ex mice than in the other groups of mice, but the alveolar septal thickness was comparable among 18W-C, 30W-C and 30W-Ex. To our interest, the sum of the Lm and septal thickness was fairly comparable between 18W-Sm (39.7 + 12.7 = 52.4 μm) and 30W-Ex (49.2 + 6.2 = 55.4 μm) in average (see Figure 1C). These measurements suggest that the increase in Lm of 30W-Ex mouse lungs resulted from normalization of the alveolar septal thickness (12.7 μm at 18 weeks → 6.2 μm at 30 weeks), which was associated with the disappearance of inflammatory cells. This further suggests that active regeneration of alveoli did not occur during 12 weeks of non-treatment after the 12-week-long smoke exposure. This defect in alveolar regeneration may be due to the continuous increase in CADM1 shedding during 12 weeks of non-treatment.

Mechanisms for the continuous increase in CADM1 shedding in 30W-Ex mice remain to be addressed. The present study identified ADAM10 as a candidate of the responsible proteases. Upregulation of ADAM10 in 30W-Ex mice did not appear to be due to active inflammation, because there are a few inflammatory cells present in these mouse lungs. Instead, we found that the αSMA level per tissue increased in 18W-Sm mice and was correlated with the ADAM10 level per epithelial cell in 30-week-old mice. Past studies reported that fibroblasts from patients with emphysema exhibit a degree of differentiation toward the myofibroblast phenotype, because they express αSMA (Hallgren et al., 2012; Karvonen et al., 2013). Upregulation of αSMA in 18W-Sm mice may be attributable to the phenotypic change of interstitial fibroblasts into myofibroblasts that was induced by the alveolar septal inflammation. On the other hand, correlation between αSMA and ADAM10 at 30 weeks may represent persistent presence of myofibroblastic cells that emerged at 18 weeks, because these cells are active in the secretion of inflammatory cytokines and mediators, including IL-1, IL-6, IL-10, TNF-α, and reactive oxygen species (Powell et al., 1999; Waghray et al., 2005; Turner et al., 2009). Interestingly, alveolar interstitial inflammation is shown to persist to a certain degree after smoking cessation. CD8 T cell oligoclonal expansions occur in ex-smoker mouse lungs (Motz et al., 2008). These mice have elevated levels of IL-10 and IL-12 in their bronchoalveolar lavage fluid (Braber et al., 2010). The persisting inflammation may be attributable, if not entirely, to the residence of interstitial myofibroblasts. In fact, we found that the αSMA level normalized to pan-cytokeratin was strongly correlated with the ADAM10 level per epithelial cell in 30-week-old mice (Figure S1C), suggesting a relative increase in myofibroblastic cells to epithelial cells as a cause of ADAM10 upregulation. Drastic remodeling of the alveolar septa seems to occur during and after the period of smoking, possibly associated with an imbalance between epithelial and myofibroblastic cells. This process may be a key event in the development and progression of emphysema in ex-smokers, because it can contribute to persisting inflammation, increased CADM1 shedding, and thereby defective alveolar regeneration.

In conclusion, we showed that emphysema progressed in 12 weeks of smoking cessation after passive smoke exposure in C57BL/6 mice, in association with continuous increase in CADM1 shedding and drastic remodeling of alveolar septa. The present study not only points out possible important role for CADM1 shedding in the development and progression of emphysema in human ex-smokers as was shown in current smokers (Mimae et al., 2014), but also suggests that remodeling of the alveolar septa that progresses during a smoke cessation period may help establish long-lasting imbalances between proteases and antiproteases, which are recognized as the central etiology of emphysema (Travis et al., 2002; Tomashefski, 2008).

## Author contributions

AR and MH carried out the animal experiments, histological examinations and Western blot analyses, and performed the statistical analyses; MH also carried out cell culture experiments; AY performed the experiments using excised mouse lungs; TI and RK participated in the Western blot analysis and histological examinations; YM provided *Cadm1*-deficient mice; AI conceived and designed the study, and drafted the manuscript. All authors read and approved the final manuscript.

### Conflict of interest statement

The authors declare that the research was conducted in the absence of any commercial or financial relationships that could be construed as a potential conflict of interest. The reviewer VS and handling Editor declared their shared affiliation.
