# Supplementary material for: Progression of Pulmonary Emphysema and Continued Increase in Ectodomain Shedding of Cell Adhesion Molecule 1 After Cessation of Cigarette Smoke Exposure in Mice
Source: Front Cell Dev Biol. 2018 May 28;6:52. doi: 10.3389/fcell.2018.00052 (PMC5985719; doi:10.3389/fcell.2018.00052)
Supplement: Supplementary file 1 [file Data_Sheet_1.pdf]

**Table S1. Measurements of suspended particulates during smoke exposure.**

| | 0.3 µm | | 0.5 µm | | 1.0 µm | |
|---|---|---|---|---|---|---|
| min | Average | SD | Average | SD | Average | SD |
| 0 | 5.937.E+06 | 2.408.E+05 | 3.383.E+05 | 1.915.E+04 | 2.426.E+04 | 2.272.E+03 |
| 15 | 4.674.E+08 | 1.551.E+07 | 4.481.E+08 | 1.369.E+07 | 3.599.E+08 | 1.515.E+07 |
| 30 | 4.978.E+08 | 2.023.E+06 | 4.659.E+08 | 7.374.E+06 | 3.332.E+08 | 2.332.E+07 |
| 45 | 4.786.E+08 | 2.801.E+06 | 3.751.E+08 | 7.382.E+06 | 3.390.E+08 | 9.062.E+06 |
| 60 | 4.681.E+08 | 5.134.E+06 | 3.653.E+08 | 4.909.E+06 | 3.398.E+08 | 2.102.E+06 |

| | 2.0 µm | | 5.0 µm | | 10.0 µm | |
|---|---|---|---|---|---|---|
| min | Average | SD | Average | SD | Average | SD |
| 0 | 2.238.E+04 | 2.353.E+03 | 0 | 0 | 0 | 0 |
| 15 | 3.563.E+08 | 1.545.E+07 | 2.466.E+05 | 1.949.E+05 | 1.126.E+04 | 1.380.E+03 |
| 30 | 3.282.E+08 | 2.376.E+07 | 1.237.E+04 | 1.540.E+03 | 2.120.E+03 | 9.349.E+02 |
| 45 | 2.988.E+08 | 5.981.E+07 | 1.979.E+05 | 1.164.E+05 | 1.084.E+04 | 1.471.E+03 |
| 60 | 2.999.E+08 | 5.693.E+07 | 1.085.E+04 | 1.153.E+03 | 5.791.E+03 | 3.789.E+02 |

The numbers of suspended particulates with indicated diameters were counted every 15 minutes after the initiation of cigarette burning, using an air particulate counter (MET ONE HHPC 6+). SD, standard deviation.

| min | Average | SD |
|---|---|---|
| 10 | 16.79 | 0.007 |
| 20 | 16.86 | 0.006 |
| 30 | 16.86 | 0.001 |
| 40 | 16.88 | 0.001 |
| 50 | 16.88 | 0.001 |
| 60 | 16.89 | 0.002 |

Concentrations of cigarette dusts (mg/m$^3$) were measured every 10 minutes after the initiation of cigarette burning, using a digital dust indicator (LD-5R). SD, standard deviation.

**Figure S1**

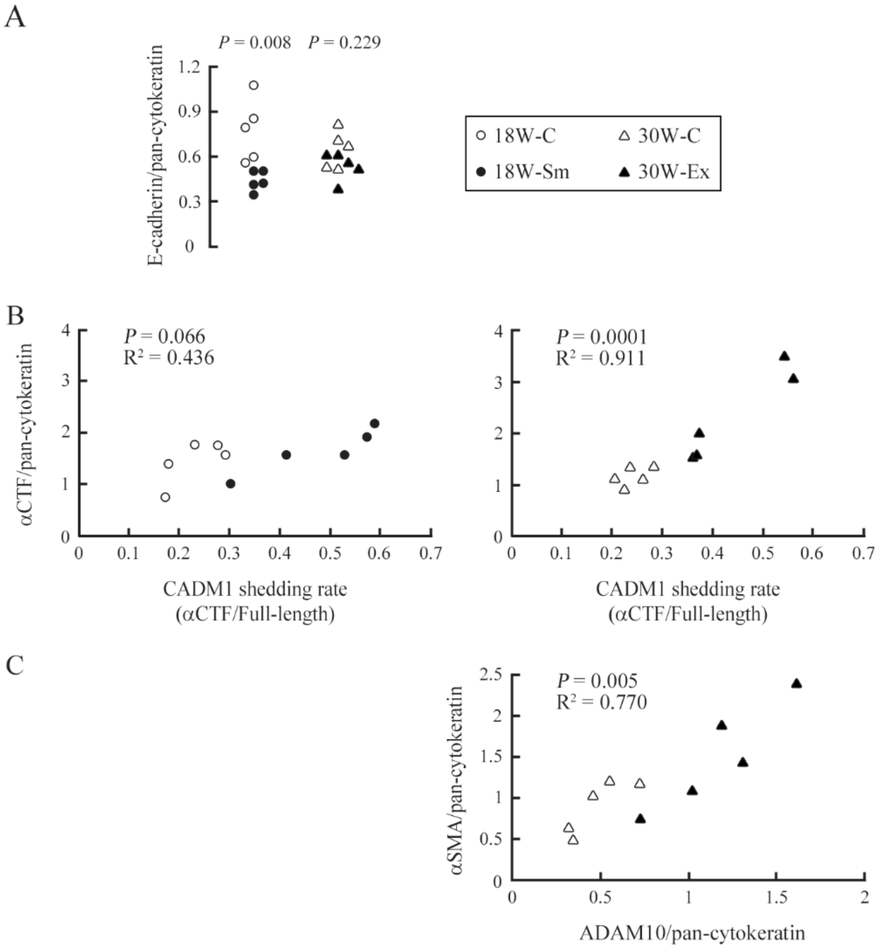


**Figure S1. Statistical analyses of the expressions of CADM1 and related molecules.** **(A)** The E-cadherin levels per lung epithelial cell (relative to pan-cytokeratin) are plotted as dots in each of the four experimental groups. Statistical significance between the two groups of the same age was analyzed using paired Student's *t*-test. *P*-values are shown. **(B)** The αCTF level per epithelial cell is shown in the scatter plots with CADM1 shedding rate. Left, 18-week-old mice (n = 10); right, 30-week-old mice (n = 10). Correlations and statistical significance were analyzed using Spearman's rank test. *P*-values and $R^2$ are shown. **(C)** The αSMA level per epithelial cell (relative to pan-cytokeratin) is scatter plotted with the ADAM10 level per epithelial cell (30-week-old

mice, n = 10). Correlation and statistical significance were analyzed using Spearman's rank test. *P*-value and $R^2$ are shown.

Figure S2

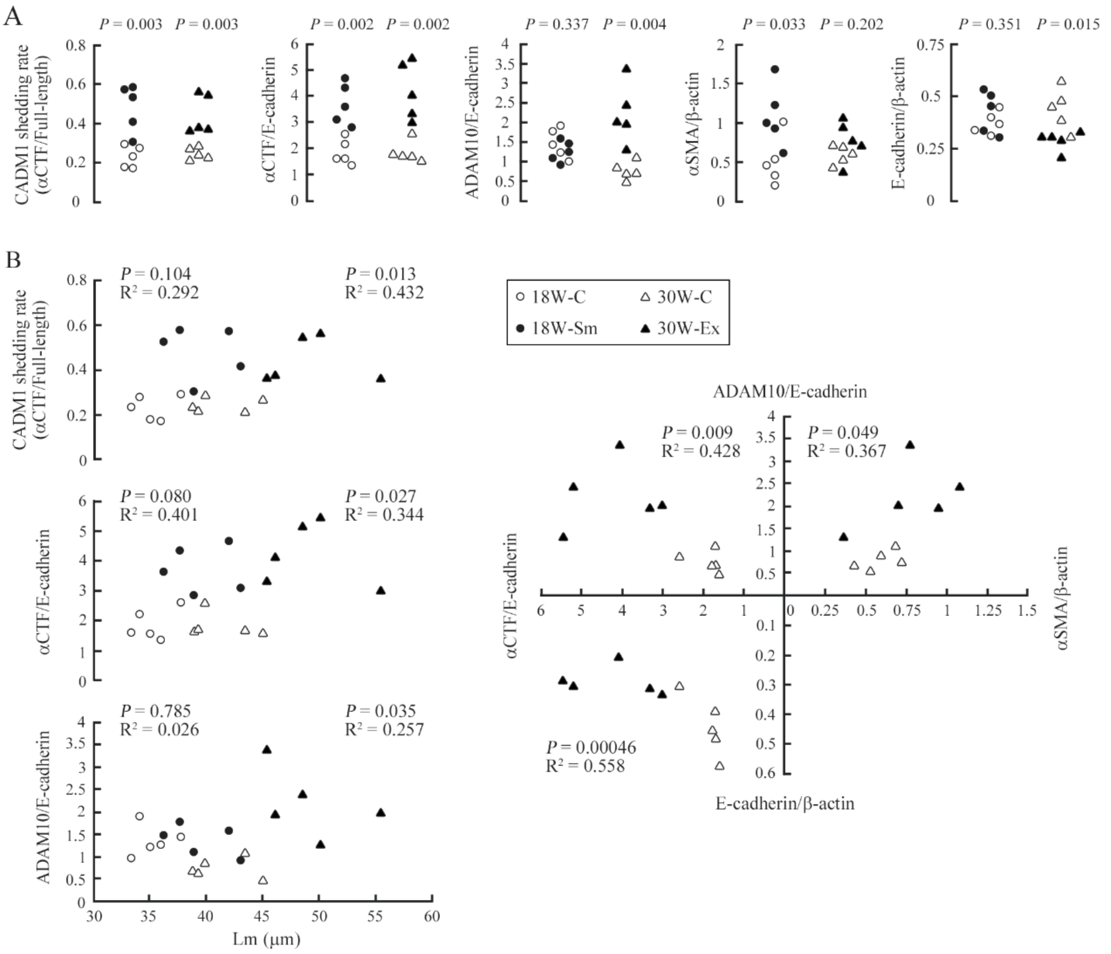


**Figure S2. Statistical analyses of the expressions of CADM1 and related molecules using E-cadherin as an epithelial marker**

**(A)** CADM1 ectodomain shedding rates (amount of αCTF relative to full-length CADM1), αCTF and ADAM10 levels per lung epithelial cell (relative to E-cadherin), and αSMA and E-cadherin levels per tissue (relative to β-actin) are plotted as dots in each group. Statistical significance between the two groups of the same age was analyzed using paired Student's *t*-test. *P*-values are shown. **(B)** The CADM1 shedding rate, αCTF level per epithelial cell, and ADAM10 level per tissue are shown in the upper three scatter plots with Lm. In the right graph, the αCTF and ADAM10 levels per

epithelial cell, and αSMA and E-cadherin levels per tissue are scatter plotted with each other. Correlations and statistical significance were analyzed using Spearman's rank test. *P*-values and $R^2$ are shown.
